# TDAE Strategy for the Synthesis of 2,3-Diaryl *N*-Tosylaziridines

**DOI:** 10.3390/molecules18077364

**Published:** 2013-06-24

**Authors:** Omar Khoumeri, Cédric Spitz, Thierry Terme, Patrice Vanelle

**Affiliations:** Laboratoire de Pharmaco-Chimie Radicalaire, Faculté de Pharmacie, Institut de Chimie Radicalaire ICR, Aix-Marseille Université, UMR CNRS 7273, 27 Boulevard Jean Moulin – CS 30064 – 13385 Marseille Cedex 05, France

**Keywords:** TDAE, *N*-tosylimines, aziridines, diastereoselectivity

## Abstract

We report herein an original and rapid synthesis of 2,3-diaryl *N*-tosylaziridines by TDAE strategy starting from *ortho*- or *para*-nitro(dichloromethyl)benzene derivatives and *N*-tosylimines. A mixture of *cis*/*trans* isomers was isolated from 1-(dichloromethyl)-4-nitrobenzene, whereas only *trans*-aziridines were obtained from *ortho*-nitro derivatives.

## 1. Introduction

Aziridines are found in a number of natural products exhibiting various biological properties, such as antitumor and antibiotic activities [1]. They are known to be valuable building blocks since they can undergo ring-opening reactions leading to a variety of amine products [2,3,4,5]. Therefore, the preparation of aziridines has received increasing attention in recent years. Various synthetic methods have been developed to prepare aziridines such as nitrene transfer to olefins [6,7,8,9,10,11], carbene addition to imines [12,13], aza-Darzens reaction [14], and ylide addition to imines [15,16].

Tetrakis(dimethylamino)ethylene (TDAE) is an organic reducing agent, which reacts with halogenated derivatives to generate a carbanion under mild conditions [17,18,19]. Since 2003, we have introduced a new program directed toward the development of original synthetic methods using TDAE methodology in medicinal chemistry [20,21,22,23,24,25,26,27]. 

In particular, we have shown that, from *o*- and *p*-nitrobenzyl chlorides, TDAE can generate a nitrobenzyl carbanion able to react with various electrophiles such as aromatic aldehydes, *α*-ketoester, ketomalonate, *α*-ketolactam, and sulfonimine derivatives [28,29,30,31].

Recently, we reported the reaction of 2-(dibromomethyl)quinoxaline and 2-(dibromomethyl)-1,4-dimethoxy-9,10-anthraquinone with aromatic aldehydes in the presence of TDAE, providing a mixture of *cis*/*trans* isomers of corresponding epoxides [32,33].

In order to extend this reactivity to the synthesis of aziridines, we explored the reaction of *gem*-dihalogenated derivatives with imines in the presence of TDAE. We chose the sulfonylaldimines for their ability to react, shown in fluorine chemistry [34] and, more recently, in anthraquinonic series [31] in the presence of TDAE. As part of our research program for new bioactive compounds [35,36,37,38], we report herein an original and efficient synthesis of 2,3-diaryl *N*-tosylaziridines using readily available *N*-tosylimines and nitro(dichloromethyl)benzene derivatives by the TDAE strategy.

## 2. Results and Discussion

**Scheme 1:**
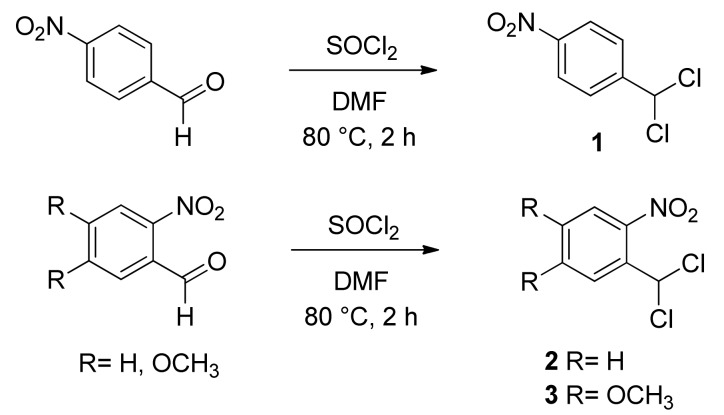
Synthesis of nitro(dichloromethyl)benzene derivatives **1**–**3**.

The required starting materials **1**–**3** were prepared in good yields (76–87%) by chlorination of the corresponding aromatic benzaldehydes using SOCl_2_ in DMF at 80 °C for 2 h (Scheme 1). Arylsubstituted *N*-tosylimines **4a**–**g** were prepared by condensation of various benzaldehydes and *p*-toluenesulfonamide in the presence of AlCl_3_ in a solvent-free procedure described by Sharghi [39].

**Scheme 2:**
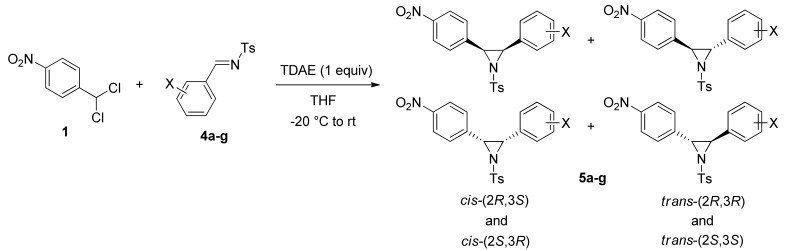
TDAE-promoted reactivity of 1-(dichloromethyl)-4-nitrobenzene (**1**) and aromatic *N*-tosylimines **4a**–**g**.

The reaction of 1-(dichloromethyl)-4-nitrobenzene **1** with two equiv. of aromatic *N*-tosylimines **4a**–**g** in the presence of TDAE at −20 °C for 1 h, followed by 2 h at rt, led to a mixture of *cis/trans* isomers of the corresponding aziridines **5a**–**g** in good yields (70–81%) as shown in Scheme 2 and reported in Table 1. Both electron-withdrawing and electron-donating substituents on the phenyl ring of the *N*-tosylimines were suitable for this reaction. ^1^H-NMR spectral studies identified the aziridines **5a**–**g** as *trans* or *cis* isomers by their coupling constant. Two distinct doublets appeared in 3.39–4.60 ppm region with *J* = 4.3–4.7 Hz or *J* = 7.3–9.4 Hz, each of the signals corresponding to one proton. The low coupling constant here is consistent with a *trans*-isomer as reported in the literature [40], higher values being indicative of the *cis*-isomer of aziridine [41].

The formation of these aziridines **5a**–**g** may be explained by nucleophilic addition of *α*-chlorocarbanion, formed by TDAE acting with 1-(dichloromethyl)-4-nitrobenzene (**1**), on the C=N double-bond of *N*-tosylimines **4a**–**g** followed by an intramolecular nucleophilic substitution. The greater stabilization of the *cis* isomer is explained by steric hindrance [15]: the largest group on the three-membered ring is the tosyl group and this will preferentially be *anti* to the other substituents to minimize 1,2-steric interactions, which forces the two remaining groups to be *cis* to each other.

**Scheme 3:**
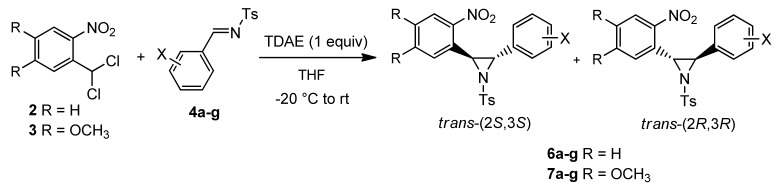
TDAE-promoted reactivity 1-(dichloromethyl)-2-nitrobenzene derivatives **2**–**3** and aromatic *N*-tosylimines **4a**–**g**.

**Scheme 4:**
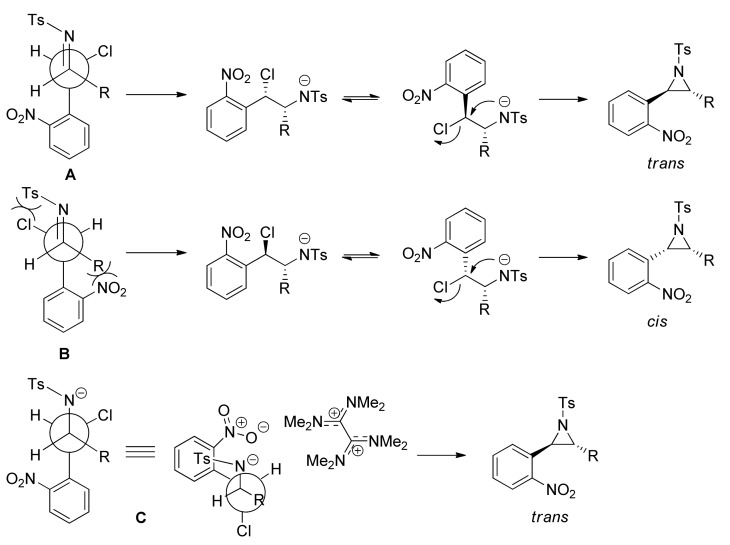
Diastereoselectivity of the aziridine formation.

The reaction of 1-(dichloromethyl)-2-nitrobenzene (**2**) and 1-(dichloromethyl)-4,5-dimethoxy-2-nitrobenzene (**3**) with two equiv. of various *N*-tosylimines **4a**–**g** in the presence of TDAE at –20 °C for 1 h followed by 2 h at rt led only to the corresponding *trans*-aziridines **6a**–**g** and **7a**–**g** in good yields (61–80%) as shown in Table 2 (Scheme 3). This total *trans* diastereoselectivy can be explained by analysing the relevant transition states (Scheme 4). The very high steric hindrance of the *ortho*-nitro subtituent of **2** and **3** with aromatic ring of sulfonimines has a significant effect. Clearly, transition state **A** is less sterically hindered than transition state **B**, which explains the preferential formation of the *trans* aziridines. To explain this total *trans* diastereoselectivity, a different coordination transition state could also be envisaged. In this hypothesis, the bis cation deriving from TDAE [42] coordinates both the TsN^−^ anion and NO_2_ group, thus stabilizing a transition state where TsN^−^ anion and NO_2_ group are on the same side like transition state **C** and increasing the formation of the *trans* aziridine that must be considered the cinetic compound.

## 3. Experimental 

### 3.1. General

Melting points were determined on a Büchi melting point B-540 apparatus and are uncorrected. Element analyses were performed on a Thermo Finnigan EA1112 at the spectropole of the Aix-Marseille University. Both ^1^H- and ^13^C-NMR spectra were determined on a Bruker AC 200 spectrometer. The ^1^H- and the ^13^C- chemical shifts are reported from CDCl_3_ peaks: ^1^H (7.26 ppm) and ^13^C (76.9 ppm). Multiplicities are represented by the following notations: s, singlet; d, doublet; t, triplet; q, quartet; m, a more complex multiplet or overlapping multiplets. The following adsorbents were used for column chromatography: silica gel 60 (Merck, particle size 0.063–0.200 mm, 70–230 mesh ASTM). TLC was performed on 5 cm × 10 cm aluminium plates coated with silica gel 60 F_254_ (Merck) in an appropriate solvent.

### 3.2. General Procedure for the Preparation of **1**–**3**

Benzaldehyde derivative (13 mmol) was dissolved in thionyl chloride (10 mL), and then to the mixture was added 1 mL of DMF. The reaction mixture was stirred for 2 h at 80 °C. Then, the solvent was removed under vacuum. The residue was dissolved in dichloromethane (100 mL), washed with H_2_O (3 × 100 mL) and dried over MgSO_4_. After evaporation, the crude product was purified by silica gel chromatography with dichloromethane: petroleum ether (1:1) to give the corresponding dichlorobenzene derivatives **1**–**3**. Analyses for compounds **1** and **2** are in agreement with those reported in the literature [43,44].

*1-(Dichloromethyl)-4,5-dimethoxy-2-nitrobenzene* (**3**). 76% yield; white solid; mp 110 °C; ^1^H-NMR (200 MHz, CDCl_3_) *δ*_H_ 3.98 (s, 3H), 4.05 (s, 3H), 7.54 (s, 1H), 7.56 (s, 1H), 7.73 (s, 1H); ^13^C-NMR (50 MHz, CDCl_3_) *δ*_C_ 56.6, 56.7, 66.4, 107.2, 110.8, 129.4, 149.8, 153.8. Anal. Calcd for C_9_H_9_Cl_2_NO_4_: C, 40.63; H, 3.41; N, 5.26. Found: C, 40.86; H, 3.26; N, 5.39.

### 3.3. General Procedure for TDAE Reaction

Into a two-necked flask equipped with a drying tube (silica gel) and a nitrogen inlet was added 15 mL of an anhydrous THF solution of dichloride derivative **1**–**3** (1 equiv.) and *N*-tosylimine **4a**–**g** (2 equiv.). The solution was cooled to −20 °C, maintained at this temperature for 30 min and then was added dropwise (via a syringe) the TDAE (1 equiv.). The solution was vigorously stirred at −20 °C for 1 h and then maintained at rt for 2 h. After this time, TLC analysis (CH_2_Cl_2_) clearly showed that compound (**1**–**3**) was totally consumed. The solution was filtered (to remove the octamethyl-oxamidinium dichloride) and hydrolyzed with H_2_O (70 mL). The aqueous solution was extracted with chloroform (3 × 40 mL), the combined organic layers washed with H_2_O (2 × 40 mL) and dried over MgSO_4_. Evaporation of the solvent furnished an orange viscous liquid as crude product. Purification by silica gel chromatography (CH_2_Cl_2_/petroleum ether: 70/30) and recrystallization from isopropanol gave corresponding aziridines (**5**–**7**). Analyses for compounds **5a**, **5d**, **5g** and **6a** are in agreement with those reported in the literature [45].

*2-(4-Nitrophenyl)-3-o-tolyl-1-tosylaziridine* (**5b**). *cis-isomer*; white solid; mp 202 °C; ^1^H-NMR (200 MHz, CDCl_3_) *δ*_H_ 2.13 (s, 3H), 2.45 (s, 3H), 4.28 (d, 1H, *J* = 7.3 Hz), 4.33 (d, 1H, *J* = 7.3 Hz), 6.91–7.14 (m, 4H), 7.22 (d, 2H, *J* = 8.6 Hz), 7.38 (d, 2H, *J* = 7.8 Hz), 7.87–7.99 (m, 4H). ^13^C-NMR (50 MHz, CDCl_3_) *δ*_C_ 21.6, 21.7, 45.6, 47.9, 123.0, 125.6, 127.9, 128.0, 128.2, 129.7, 129.8, 130.0, 131.5, 134.4, 134.5, 135.9, 139.6, 145.2. *trans-isomer*; white solid; mp 161 °C; ^1^H-NMR (200 MHz, CDCl_3_) *δ*_H_ 2.38 (s, 3H), 2.41 (s, 3H), 4.20 (d, 1H, *J* = 4.7 Hz), 4.35 (d, 1H, *J* = 4.7 Hz), 7.17–7.28 (m, 6H), 7.59–7.66 (m, 4H), 8.21 (d, 2H, *J* = 8.7 Hz). ^13^C-NMR (50 MHz, CDCl_3_) *δ*_C_ 18.8, 21.6, 45.6, 47.8, 123.0, 125.6, 127.9, 128.0, 128.1, 128.1, 129.0, 129.7, 129.9, 134.3, 135.9, 139.5, 145.2, 147.3. Anal. Calcd for C_22_H_20_N_2_O_4_S: C, 64.69; H, 4.94; N, 6.86; S, 7.85. Found: C, 64.79; H, 4.97; N, 6.85; S, 7.92.

*2-(2-Chlorophenyl)-3-(4-nitrophenyl)-1-tosylaziridine* (**5c**). *cis-isomer*; white solid; mp 193 °C; ^1^H-NMR (200 MHz, CDCl_3_) *δ*_H_ 2.45 (s, 3H), 3.39 (d, 1H, *J* = 7.6 Hz), 3.46 (d, 1H, *J* = 7.6 Hz), 7.04–7.20 (m, 4H), 7.26 (d, 2H, *J* = 8.6 Hz), 7.38 (d, 2H, *J* = 8.2 Hz), 7.93 (d, 2H, *J* = 8.6 Hz), 7.97 (d, 2H, *J* = 8.2 Hz). ^13^C-NMR (50 MHz, CDCl_3_) *δ*_C_ 21.6, 46.0, 46.9, 123.1, 126.5, 128.0, 128.3, 129.0, 129.3, 129.4, 129.5, 130.0, 133.2, 134.1, 139.1, 145.3, 147.4. *trans-isomer*; white solid; mp 185 °C; ^1^H-NMR (200 MHz, CDCl_3_) *δ*_H_ 2.42 (s, 3H), 4.10 (d, 1H, *J* = 4.5 Hz), 4.56 (d, 1H, *J* = 4.5 Hz), 7.22–7.41 (m, 6H), 7.69 (d, 2H, *J* = 8.7 Hz), 7.73 (d, 2H, *J* = 8.7 Hz), 8.23 (d, 2H, *J* = 8.7 Hz). ^13^C-NMR (50 MHz, CDCl_3_) *δ*_C_ 21.6, 47.2, 49.8, 123.5, 127.0, 127.7, 128.4, 129.4, 129.6, 130.0, 130.0, 131.2, 134.5, 136.1, 139.5, 144.8, 148.1. Anal. Calcd for C_21_H_17_ClN_2_O_4_S: C, 58.81; H, 4.00; N, 6.53; S, 7.48. Found: C, 58.88; H, 3.99; N, 6.43; S, 7.49.

*2-(3-Fluorophenyl)-3-(4-nitrophenyl)-1-tosylaziridine* (**5e**). *cis-isomer*; white solid; mp 108 °C; ^1^H-NMR (200 MHz, CDCl_3_) *δ*_H_ 2.47 (s, 3H), 4.22 (d, 1H, *J* = 9.4Hz), 4.32 (d, 1H, *J* = 9.4Hz), 6.69–6.88 (m, 3H), 7.04–7.16 (m, 1H), 7.23 (d, 2H, *J* = 8.3 Hz), 7.39 (d, 2H, *J* = 8.3 Hz), 7.96 (d, 2H, *J* = 8.3 Hz) 7.99 (d, 2H, *J* = 8.3 Hz). ^13^C-NMR (50 MHz, CDCl_3_) *δ*_C_ 21.7, 46.3, 47.1 (d, *J* = 2.6 Hz), 114.5 (d, *J* = 22.7 Hz), 115.2 (d, *J* = 21.1 Hz) 123.2 (d, *J* = 2.9 Hz), 123.3, 128.0, 128.5, 129.9, 130.1, 133.7 (d, *J* = 8.0 Hz), 134.2, 139.1, 145.4, 147.6, 162.4 (d, *J* = 247.0 Hz). *trans-isomer*; white solid; mp 143 °C; ^1^H-NMR (200 MHz, CDCl_3_) *δ*_H_ 2.41 (s, 3H), 4.22 (d, 1H, *J* = 4.4 Hz), 4.26 (d, 1H, *J* = 4.4 Hz), 7.70 (d, 2H, *J* = 8.8 Hz), 7.24–7.41 (m, 4H), 7.60 (d, 2H, *J* = 8.8 Hz), 7.67 (d, 2H, *J* = 8.2 Hz), 8.21 (d, 2H, *J* = 8.8 Hz). ^13^C NMR (50 MHz, CDCl_3_) *δ*_C_ 21.6, 49.0, 50.1 (d, *J* = 2.2 Hz), 115.2 (d, *J* = 22.7 Hz), 116.1 (d, *J* = 21.2 Hz), 123.7, 124.0 (d, *J* = 2.9 Hz), 127.5, 129.2, 129.7, 130.0, 134.7 (d, *J* = 7.7 Hz), 136.4, 140.2, 144.7, 148.1, 162.7 (d, *J* = 247.4Hz). Anal. Calcd for C_21_H_17_FN_2_O_4_S: C, 61.16; H, 4.15; N, 6.79; S, 7.77. Found: C, 60.51; H, 4.19; N, 6.62; S, 7.66.

*2-(4-Nitrophenyl)-3-(3-(trifluoromethyl)phenyl)-1-tosyl-aziridine* (**5f**). *cis-isomer*; white solid; mp 63 °C; ^1^H-NMR (200 MHz, CDCl_3_) *δ*_H_ 2.44 (s, 3H), 4.30 (d, 1H, *J* = 7.7 Hz), 4.34 (d, 1H, *J* = 7.7 Hz), 7.21–7.41 (m, 8H), 7.95 (d, 2H, *J* = 8.4 Hz), 7.99 (d, 2H, *J* = 8.4 Hz). ^13^C-NMR (50 MHz, CDCl_3_) *δ*_C_ 21.6, 46.4, 46.9, 123.3, 124.4 (q, *J* = 4.0 Hz), 125.0 (q, *J* = 4.0 Hz), 128.0, 128.5, 128.8, 130.0, 130.5 (q, *J* = 33.0 Hz), 130.7, 132.3, 133.9, 138.9, 142.5 (q, *J* = 238.9 Hz), 145.6, 147.5. *trans-isomer*; white solid; mp 164 °C; ^1^H-NMR (200 MHz, CDCl_3_) *δ*_H_ 2.40 (s, 3H), 4.25 (d, 1H, *J* = 4.3 Hz), 4.35 (d, 1H, *J* = 4.3 Hz), 7.20–7.25 (m, 4H), 7.54–7.65 (m, 4H), 7.97 (d, 2H, *J* = 8.8 Hz), 8.20 (d, 2H, *J* = 8.8 Hz). ^13^C-NMR (50 MHz, CDCl_3_) *δ*_C_ 21.5, 48.5, 50.1, 123.7, 125.3 (q, *J* = 3.7Hz), 125.8 (q, *J* = 3.7 Hz), 127.5, 129.1, 129.7, 130.1, 130.9 (q, *J* = 32.6 Hz), 131.6, 133.1, 136.1, 140.1, 140.5 (q, *J* = 238.1 Hz), 144.9, 148.1. Anal. Calcd for C_22_H_17_F_3_N_2_O_4_S: C, 57.14; H, 3.71; N, 6.06; S, 6.93. Found: C, 55.46; H, 3.74; N, 5.92; S, 6.71.

*trans-2-(2-Nitrophenyl)-3-o-tolyl-1-tosylaziridine* (**6b**). White solid; mp 160 °C; ^1^H-NMR (200 MHz, CDCl_3_) *δ*_H_ 2.27 (s, 3H), 2.41 (s, 3H), 3.87 (d, 1H, *J* = 4.8 Hz), 5.16 (d, 1H, *J* = 4.8 Hz), 7.16–7.20 (m, 4H), 7.26–7.32 (m, 1H), 7.48–7.77 (m, 6H), 8.15 (dd, 1H, *J* = 8.1, 1.1 Hz). ^13^C-NMR (50 MHz, CDCl_3_) *δ*_C_ 19.3, 21.5, 43.6, 51.9, 124.9, 125.7, 127.9, 128.5, 128.6, 129.1, 129.2, 129.4, 129.7, 129.8, 131.4, 134.2, 135.4, 139.6, 144.3, 148.1. Anal. Calcd for C_22_H_20_N_2_O_4_S: C, 64.69; H, 4.94; N, 6.86; S, 7.85. Found: C, 64.81; H, 4.96; N, 6.82; S, 7.57.

*trans-2-(2-Chlorophenyl)-3-(2-nitrophenyl)-1-tosyl-aziridine* (**6c**). White solid; mp 153 °C; ^1^H-NMR (200 MHz, CDCl_3_) *δ*_H_ 2.42 (s, 3H), 4.25 (d, 1H, *J* = 4.8 Hz), 5.04 (d, 1H, *J* = 4.8 Hz), 7.21–7.35 (m, 5H), 7.51–7.72 (m, 5H), 7.87 (d, 1H, *J* = 7.6 Hz), 8.20 (d, 1H, *J* = 7.6 Hz). ^13^C-NMR (50 MHz, CDCl_3_) *δ*_C_ 21.6, 46.0, 49.3, 125.0, 126.4, 127.8, 129.1, 129.5, 129.6, 129.9, 130.0, 130.1, 130.2, 130.3, 134.2, 135.7, 136.0, 144.6, 148.5. Anal. Calcd for C_21_H_17_ClN_2_O_4_S: C, 58.81; H, 4.00; N, 6.53; S, 7.48. Found: C, 58.72; H, 3.99; N, 6.50; S, 7.46.

*trans-2-(2-Bromophenyl)-3-(2-nitrophenyl)-1-tosyl-aziridine* (**6d**). White solid; mp 153 °C; ^1^H-NMR (200 MHz, CDCl_3_) *δ*_H_ 2.41 (s, 3H), 4.26 (d, 1H, *J* = 4.9 Hz), 5.00 (d, 1H, *J* = 4.9 Hz), 7.21–7.26 (m, 2H), 7.29–7.42 (m, 2H), 7.51–7.72 (m, 6H), 7.89–7.92 (m, 1H), 8.18 (dd, 1H, *J* = 8.1 Hz, *J* = 1.0 Hz). ^13^C-NMR (50 MHz, CDCl_3_) *δ*_C_ 21.5, 46.4, 51.1, 125.0, 127.3, 127.8, 128.1, 129.4, 129.6, 129.7, 129.8, 130.0, 130.3, 131.3, 132.3, 134.1, 135.6, 144.6, 148.5. Anal. Calcd for C_21_H_17_BrN_2_O_4_S: C, 53.29; H, 3.62; N, 5.92; S, 6.77. Found: C, 53.36; H, 3.66; N, 5.96; S, 6.78.

*trans-2-(3-Fluorophenyl)-3-(2-nitrophenyl)-1-tosyl-aziridine* (**6e**). White solid; mp 154 °C; ^1^H-NMR (200 MHz, CDCl_3_) *δ*_H_ 2.42 (s, 3H), 3.91 (d, 1H, *J* = 4.6 Hz), 5.03 (d, 1H, *J* = 4.6 Hz), 7.01–7.26 (m, 4H), 7.31–7.36 (m, 2H), 7.48–7.69 (m, 5H), 8.17 (d, 1H, *J* = 7.9 Hz). ^13^C-NMR (50 MHz, CDCl_3_) *δ*_C_ 21.6, 45.5, 51.6 (d, *J* = 2.2 Hz), 116.1 (d, *J* = 20.8 Hz), 116.5 (d, *J* = 22.7 Hz), 125.0, 125.3 (d, *J* = 2.9 Hz), 127.8, 129.4, 129.5, 129.8, 129.9, 130.5, 133.1 (d, *J* = 8.0 Hz), 134.2, 135.8, 144.6, 148.2, 162.4 (d, *J* = 246.6 Hz). Anal. Calcd for C_21_H_17_FN_2_O_4_S: C, 61.16; H, 4.15; N, 6.79; S, 7.77. Found: C, 61.29; H, 4.20; N, 6.75; S, 7.72.

*trans-2-(2-Nitrophenyl)-3-(3-(trifluoromethyl)phenyl)-aziridine* (**6f**). White solid; mp 145 °C; ^1^H-NMR (200 MHz, CDCl_3_) *δ*_H_ 2.41 (s, 3H), 3.91 (d, 1H, *J* = 4.5 Hz), 5.13 (d, 1H, *J* = 4.5 Hz), 7.21 (d, 2H, *J* = 8.1 Hz), 7.49–7.84 (m, 9H), 8.18 (d, 1H, *J* = 8.4 Hz). ^13^C-NMR (50 MHz, CDCl_3_) *δ*_C_ 21.5, 44.9, 51.6, 121.5 (q, *J* = 272.2 Hz), 125.0, 125.8 (q, *J* = 3.7 Hz), 126.8 (q, *J* = 3.7 Hz), 127.7, 128.9, 129.5, 129.6, 129.8, 130.4 (q, *J* = 32.2 Hz), 130.6, 131.5, 132.8, 134.3, 135.5, 144.8, 148.1. Anal. Calcd for C_22_H_17_F_3_N_2_O_4_S: C, 57.14; H, 3.71; N, 6.06; S, 6.93. Found: C, 56.96; H, 3.72; N, 6.11; S, 6.72.

*trans-2-(4-Fluorophenyl)-3-(2-nitrophenyl)-1-tosylaziridine* (**6g**). White solid; mp 135 °C; ^1^H-NMR (200 MHz, CDCl_3_) *δ*_H_ 2.42 (s, 3H), 3.86 (d, 1H, *J* = 4.6 Hz), 5.10 (d, 1H, *J* = 4.6 Hz), 7.04 (t, 2H, *J* = 8.4 Hz), 7.23 (t, 2H, *J* = 8.4 Hz), 7.47–7.63 (m, 7H), 8.15 (d, 1H, *J* = 7.8 Hz). ^13^C-NMR (50 MHz, CDCl_3_) *δ*_C_ 21.6, 44.9, 52.4, 115.3 (d, *J* = 21.6 Hz), 125.0, 126.2 (d, *J* = 3.3 Hz), 127.7, 129.3, 129.5, 129.6, 131.1, 131.7 (d, *J* = 8.4 Hz), 134.3, 136.0, 144.5, 148.1, 162.5 (d, *J* = 248.4 Hz). Anal. Calcd for C_21_H_17_FN_2_O_4_S: C, 61.16; H, 4.15; N, 6.79; S, 7.77. Found: C, 61.31; H, 4.20; N, 6.79; S, 7.71.

*trans-2-(4,5-Dimethoxy-2-nitrophenyl)-3-phenyl-1-tosylaziridine* (**7a**). White solid; mp 154 °C; ^1^H-NMR (200 MHz, CDCl_3_) *δ*_H_ 2.39 (s, 3H), 3.74 (s, 3H), 3.88 (d, 1H, *J* = 4.4 Hz), 3.94 (s, 3H), 5.15 (d, 1H, *J* = 4.4 Hz), 6.91 (s, 1H), 7.19–7.23 (m, 2H), 7.34–7.37 (m, 3H), 7.59–7.63 (m, 4H), 7.71 (s, 1H). ^13^C-NMR (50 MHz, CDCl_3_) *δ*_C_ 21.5, 45.3, 53.7, 56.1, 56.4, 107.8, 110.6, 126.1, 127.8, 128.2, 129.0, 129.5, 129.9, 130.2, 136.5, 140.3, 144.2, 148.5, 153.7. Anal. Calcd for C_23_H_22_N_2_O_6_S: C, 60.78; H, 4.88; N, 6.16; S, 7.06. Found: C, 60.80; H, 4.92; N, 6.20; S, 7.03.

*trans-2-(4,5-Dimethoxy-2-nitrophenyl)-3-o-tolyl-1-tosylaziridine* (**7b**). White solid; mp 167 °C; ^1^H-NMR (200 MHz, CDCl_3_) *δ*_H_ 2.38 (s, 6H), 3.76 (s, 3H), 3.82 (d, 1H, *J* = 4.8 Hz), 3.93 (s, 3H), 5.18 (d, 1H, *J* = 4.8 Hz), 6.98 (s, 1H), 7.18 (d, 4H, *J* = 7.3 Hz), 7.24–7.32 (m, 2H), 7.56 (d, 1H, *J* = 8.2 Hz), 7.65 (d, 1H, *J* = 7.3 Hz), 7.70 (s, 1H). ^13^C-NMR (50 MHz, CDCl_3_) *δ*_C_ 19.4, 21.4, 44.3, 52.5, 56.1, 56.4, 107.8, 110.7, 125.7, 126.4, 127.9, 128.5, 128.7, 129.2, 129.4, 129.8, 135.9, 139.9, 140.2, 144.2, 148.4, 153.7. Anal. Calcd for C_24_H_24_N_2_O_6_S: C, 61.52; H, 5.16; N, 5.98; S, 6.84. Found: C, 61.86; H, 5.21; N, 5.98; S, 6.78.

*trans-2-(2-Chlorophenyl)-3-(4,5-dimethoxy-2-nitrophenyl)-1-tosylaziridine* (**7c**). White solid; mp 144 °C; ^1^H-NMR (200 MHz, CDCl_3_) *δ*_H_ 2.41 (s, 3H), 3.83 (s, 3H), 3.95 (s, 3H), 4.17 (d, 1H, *J* = 4.9 Hz), 5.07 (d, 1H, *J* = 4.9 Hz), 7.09 (s, 1H), 7.24 (d, 2H, *J* = 8.2 Hz), 7.29–7.40 (m, 3H), 7.65 (d, 2H, *J* = 8.2 Hz), 7.72–7.76 (m, 2H).^13^C-NMR (50 MHz, CDCl_3_) *δ*_C_ 21.5, 46.3, 50.0, 56.2, 56.4, 107.9, 111.3, 125.0, 126.8, 127.9, 129.1, 129.4, 129.5, 130.1, 130.3, 136.0, 136.4, 140.8, 144.5, 148.8, 153.6. Anal. Calcd for C_23_H_21_ClN_2_O_6_S: C, 56.50; H, 4.33; N, 5.73; S, 6.56. Found: C, 56.44; H, 4.33; N, 5.71; S, 6.57.

*trans-2-(2-Bromophenyl)-3-(4,5-dimethoxy-2-nitrophenyl)-1-tosylaziridine* (**7d**). White solid; mp 164 °C; ^1^H-NMR (200 MHz, CDCl_3_) *δ*_H_ 2.40 (s, 3H), 3.85 (s, 3H), 3.94 (s, 3H), 4.19 (d, 1H, *J* = 4.8 Hz), 5.02 (d, 1H, *J* = 4.8 Hz), 7.14 (s, 1H), 7.21–7.25 (m, 3H), 7.34 (t, 2H, *J* = 7.3 Hz), 7.53–7.72 (m, 3H), 7.74 (s, 1H). ^13^C-NMR (50 MHz, CDCl_3_) *δ*_C_ 21.5, 47.0, 51.7, 56.3, 56.4, 107.9, 111.6, 124.6, 126.3, 127.4, 127.9, 129.5, 130.1, 130.4, 131.2, 132.3, 135.9, 140.9, 144.5, 148.8, 153.4. Anal. Calcd for C_23_H_21_BrN_2_O_6_S: C, 51.79; H, 3.97; N, 5.25; S, 6.01. Found: C, 51.77; H, 3.93; N, 5.22; S, 5.88.

*trans-2-(4,5-Dimethoxy-2-nitrophenyl)-3-(3-fluorophenyl)-1-tosylaziridine* (**7e**). White solid; mp 161 °C; ^1^H-NMR (200 MHz, CDCl_3_) *δ*_H_ 2.41 (s, 3H), 3.75 (s, 3H), 3.84 (d, 1H, *J* = 4.4 Hz), 3.95 (s, 3H), 5.08 (d, 1H, *J* = 4.4 Hz), 6.91 (s, 1H), 7.03–7.12 (m, 1H), 7.23–7.29 (m, 3H), 7.33–7.45 (m, 2H), 7.65 (d, 2H, *J* = 8.3 Hz), 7.72 (s, 1H). ^13^C-NMR (50 MHz, CDCl_3_) *δ*_C_ 21.6, 45.7, 52.7, 56.2, 56.5, 107.9, 110.7, 116.2 (d, *J* = 20.8 Hz), 116.9 (d, *J* = 22.7 Hz), 125.7, 127.9, 129.5, 129.6, 129.8 (d, *J* = 8.0 Hz), 132.9 (d, *J* = 8.0 Hz), 136.3, 144.6, 148.7, 153.8, 160.0, 162.5 (d, *J* = 248.6 Hz). Anal. Calcd for C_23_H_21_FN_2_O_6_S: C, 58.47; H, 4.48; N, 5.93; S, 6.79. Found: C, 58.55; H, 4.54; N, 5.92; S, 6.76.

*trans-2-(4,5-Dimethoxy-2-nitrophenyl)-3-(3-(trifluoromethyl)phenyl)-1-tosylaziridine* (**7f**). White solid; mp 163 °C; ^1^H-NMR (200 MHz, CDCl_3_) *δ*_H_ 2.39 (s, 3H), 2.76 (s, 3H), 3.85 (d, 1H, *J* = 4.5 Hz), 3.93 (s, 3H), 5.14 (d, 1H, *J* = 4.5 Hz), 6.94 (s, 1H), 7.21 (d, 2H, *J* = 8.1 Hz), 7.48–7.68 (m, 5H), 7.71 (s, 1H), 7.88 (d, 1H, *J* = 7.4 Hz). ^13^C-NMR (50 MHz, CDCl_3_) *δ*_C_ 21.4, 45.3, 52.3, 56.2, 56.4, 107.8, 110.6, 125.5 (q, *J* = 272.6 Hz), 125.8 (q, *J* = 3.7 Hz), 126.9 (q, *J* = 4.0 Hz), 127.7, 128.8, 128.8, 129.6, 130.4 (q, *J* = 32.6 Hz), 131.3, 133.1, 135.9, 140.2, 144.7, 148.7, 153.8. Anal. Calcd for C_24_H_21_F_3_N_2_O_6_S: C, 55.17; H, 4.05; N, 5.36; S, 6.14. Found: C, 55.21; H, 4.19; N, 5.41; S, 6.05.

*trans-2-(4,5-Dimethoxy-2-nitrophenyl)-3-(4-fluorophenyl)-1-tosylaziridine* (**7g**). White solid; mp 158 °C; ^1^H-NMR (200 MHz, CDCl_3_) *δ*_H_ 2.41 (s, 3H), 3.72 (s, 3H), 3.82 (d, 1H, *J* = 4.4 Hz), 3.90 (s, 3H), 5.13 (d, 1H, *J* = 4.4 Hz), 6.86 (s, 1H), 7.06 (t, 2H, *J* = 8.6 Hz), 7.23–7.28 (m, 2H), 7.58–7.78 (m, 5H). ^13^C-NMR (50 MHz, CDCl_3_) *δ*_C_ 21.5, 45.5, 53.1, 56.1, 56.4, 107.9, 110.5, 115.3 (d, *J* = 21.6 Hz), 126.1 (d, *J* = 2.2 Hz), 126.2, 127.8, 129.6, 131.9 (d, *J* = 8.4 Hz), 136.6, 140.3, 144.4, 148.6, 153.8, 162.8 (d, *J* = 248.8 Hz). Anal. Calcd for C_23_H_21_FN_2_O_6_S: C, 58.47; H, 4.48; N, 5.93; S, 6.79. Found: C, 58.53; H, 4.51; N, 5.90; S, 6.62.

## 4. Conclusions 

TDAE methodology is extended here to the reaction of *ortho*- or *para*-nitro dichloromethylbenzene derivatives **1**–**3** with various aromatic *N*-tosylimines **4a**–**g**, leading to the corresponding aziridines **5**–**7** in good yields (61–81%). The diastereoselectivity of the reaction is shown to be sensitive to steric hindrance. Further research is in progress to extent this method to other dichloride derivatives and to explore the ring opening reactions of the aziridines.

## Figures and Tables

**Table 1:** Reaction of 1-(dichloromethyl)-4-nitrobenzene (**1)** with aromatic *N*-tosylimines **4a**–**g** using TDAE strategy. ^a^

Entry	X	Aziridine	*cis*/*trans* isomers ^b^ (%)	Yield^c^ (%)
1	H	5a	86/14	81
2	2-Me	5b	67/33	74
3	2-Cl	5c	74/26	70
4	2-Br	5d	68/32	72
5	3-F	5e	86/14	71
6	3-CF_3_	5f	75/25	73
7	4-F	5g	84/16	80

^a^ All the reactions were performed using two equiv. of sulfonimines **4a**–**g**, one equiv. of dichloride **1** and one equiv. of TDAE in anhydrous THF at –20 °C for 1 h and then at rt for 2 h. ^b^ Determined by ^1^H-NMR of the crude product. ^c^ All yields refer to chromatographically isolated pure products and are relative to dichloride **1**.

**Table 2:** Reaction of 1-(dichloromethyl)-2-nitrobenzene derivatives **2**–**3** with aromatic *N*-tosylimines **4a**–**g** using TDAE strategy. ^a^

Entry	Substrate	X	*trans*-Aziridine ^b^	Yield ^c^ (%)
1	2	H	6a	70
2	2	2-Me	6b	62
3	2	2-Cl	6c	80
4	2	2-Br	6d	70
5	2	3-F	6e	75
6	2	3-CF_3_	6f	63
7	2	4-F	6g	79
8	3	H	7a	73
9	3	2-Me	7b	70
10	3	2-Cl	7c	61
11	3	2-Br	7d	74
12	3	3-F	7e	68
13	3	3-CF_3_	7f	75
14	3	4-F	7g	64

^a^ All the reactions were performed using 2 equiv of sulfonimines **4a**–**g**, 1 equiv of dichloride **2**–**3** and 1 equiv of TDAE in anhydrous THF at –20 °C for 1 h and then at rt for 2 h. ^b^ Determined by ^1H-NMR^ of the crude product. ^c^ All yields refer to chromatographically isolated pure products and are relative to dichloride **2**–**3**.
